# Study on pumping wear characteristics of concrete pipeline based on CFD-DEM coupling

**DOI:** 10.1038/s41598-023-42995-1

**Published:** 2023-09-26

**Authors:** Yuankun Liao, Kai Cheng, Wuhe Sun, Yan Zhao

**Affiliations:** School of Mechanical and AerosPace Engineering, Jilin University, Changchun, 130022 Jilin China

**Keywords:** Civil engineering, Theory and computation

## Abstract

Concrete pumping, integral to building construction, governs pipeline durability and overall construction efficiency. This study advances the traditional methods by employing the CFD-DEM coupling technique, a pioneering approach that integrates the irregular shape of coarse aggregates, capturing the intricacies of concrete pumping in pipelines. Beyond identifying primary wear areas and quantifying wear magnitudes, this research unveils a quadratic progression in average wear over time. The most pronounced wear appears in the pipeline’s bent sections. Notably, pumping speed, more influential than aggregate volume fraction, is pivotal to pipeline wear. Minimized speeds increase blockage risks, notably at elbows, while augmented speeds intensify wear. An optimal speed range between 2 and 3 m/s is deduced. Additionally, as the aggregate volume fraction surges, wear amplifies and blockages frequent. Hence, an aggregate volume fraction between 15 and 20% emerges as the recommendation.

## Introduction

With the rapid development of infrastructure construction in China, the concrete pump truck has a series of functions such as high construction efficiency, drivability, and pumping^1–3^. Therefore, it is widely used in the construction fields such as houses, bridges, and roads. However, with the escalating utilization of concrete pump trucks and pipelines, certain challenges associated with pipeline wear have emerged^4–6^. In the process of concrete transportation, the long-term impact of aggregate will lead to the easy wear of the transportation pipeline. The light wear of the pipeline will lead to concrete leakage, while the heavy wear will affect the pumping efficiency and safety of the whole transportation pipeline. Meanwhile, in the process of concrete transportation, it will inevitably lead to the loss of pumping pressure. If the pressure loss is large, it will easily lead to serious problems such as bleeding and segregation, and coarse aggregate congestion^7–9^. Therefore, the research on concrete pumping has important theoretical significance and practical values.

For concrete pipeline transportation, many researchers at home and abroad have done many experiments. Ye^10^ constructed a dynamic monitoring system for the stress and strain of the pumping pipeline, and continuous experimental monitoring is carried out on the strain data of the pipeline under real pumping conditions, and the dynamic strain data of each measuring point of the pipeline are obtained. The results indicated that the circumferential strain and axial strain of the pipeline show obvious periodic changes with the piston stroke movement, and the circumferential strain is significantly greater than the axial strain, and the circumferential strain and axial strain gradually decrease with the pumping length extension. Chen et al.^11^ tested the working performance and viscosity change of concrete in a super-long pipeline by horizontal coil, and the pressure change along the pipeline was also detected. The results showed that the slump spread of self-compacting concrete before and after pumping through the horizontal pipeline lost 12 cm, the plastic viscosity decreased by 82%, the yield stress increased by 86%, and the average pressure drop in the horizontal pipeline was 0.012 MPa/m. Jacobsen et al.^12^ explored the flow changes of different materials (rubber, steel, acrylic acid) on the cross section of the pipeline are investigated. The results show that the flow velocity is the largest near the center of the pipeline, while the flow velocity at the wall of the pipeline is lower. Wu et al.^13^ developed field detection equipment for concrete pumping viscosity resistance, which can quickly and accurately detect the pumping resistance of concrete. The results show that the higher the label of high-strength and high-performance concrete, the greater its adhesion coefficient and velocity coefficient, indicating that the greater the pressure required for concrete to flow from a static state in the pipeline. In addition, the greater the friction resistance between concrete and the pipe wall with the increase in pumping speed, which provides a basis for the optimal layout of the transportation pipeline. Wu et al.^14^ explored the influence of silica fume, admixture, and fiber lamp components on concrete pumping through experiments. The results show that the increase in steel fiber infiltration obviously affects the working performance of high-strength concrete. The plastic viscosity of ultra-high-strength concrete mixture decreases with the increase of bead infiltration, and the effect of the viscosity reducer is not as good as that of high-permeability beads, which provides technical guidance for the high-performance design of high-strength concrete and promotes the large-scale application process of high-strength pumping concrete.

Due to the development of computer technology and the improvement of numerical algorithms, many researchers also use numerical simulation to study the fluidity of concrete. Wu et al.^15^ employed the SPH (smoothed particle hydrodynamics) approach to simulate the rheology of concrete. SPH, an advanced computational fluid dynamics method, relies on inter-particle interactions rather than a fixed computational grid, making it apt for simulating fluid flows with intricate boundaries and significant deformations. In his study, both the slump test and L-box test of concrete were simulated, with the Bingham model serving as the material constitutive model for concrete. The close alignment between the simulated outcomes and experimental results underscores the feasibility of this numerical method in assessing the flow properties of concrete. Tan et al.^16^ established the discrete element model and predicted the distribution of pipe wall wear. The results show that the pipe wall wear increases rapidly with the increase of pumping speed and steadily with the increase of elbow angle. Based on the on-site coil pumping experiment, Li et al.^17^ simplified the concrete into two-phase flow, and the flow characteristics of concrete in the conveying pipe are simulated, and compared with the experimental results and theoretical calculation results of the coil. It is found that the pressure loss value is basically consistent, which verifies the effectiveness of the computer numerical simulation results. Using PFC2D software, Wang^18^ simulated the pressure loss of concrete in different conveying pipes by discrete element method, and the movement law and stress characteristics of concrete are studied. The findings show that the numerical simulation results of horizontal straight pipe are different from those of code, but the law is consistent, and the numerical simulation results of conical pipe are 2–3 times that of horizontal straight pipe. Yuan and Guo^19^ proposed a new elliptical particle model of concrete. Jiang et al.^20^ employed the CFD-DEM coupling technique to simulate the pumping process of fresh concrete in various pipelines. This method utilizes CFD to represent fluid dynamics and adopts DEM to model solid behavior, rendering it suitable for analyzing intricate flow regimes where particles and fluids are concurrently present. He also investigated the influence of varying pipeline structural Parameters and inclination angles on pressure loss. Taking the minimum pressure loss as the goal, the objective function of pressure loss is established, and an optimization method based on the CFD-DEM coupling model and the single objective optimization algorithm is proposed. The results show that the pressure loss of the pump truck conveying pipe is reduced by 5.45% and the average velocity of coarse aggregate at the outlet of the pipeline is also reduced under the optimization model.

To summarize, given the diversity of concrete types and the complexity of its constituents, existing studies employing numerical simulations for concrete pipeline pumping often overlook the rheological properties of concrete or the irregularities in aggregate shape, frequently relying solely on either two-phase flow or discrete element methods for simulations. While multiphase flow methodologies have been extensively adopted, they still face challenges, particularly in scenarios with high solid volume fractions, especially in capturing intricate interactions between particles and between particles and the liquid phase. Moreover, when applied standalone, the discrete element method (DEM) exhibits a pronounced sensitivity to model parameters; even minor parameter deviations can compromise simulation stability. A review of the current literature reveals that many studies focus on pressure loss in pipeline pumping, with a paucity of systematic investigations into wear issues or the effects of specific factors on wear. Accordingly, this study employs a CFD-DEM coupled approach, a novel methodology that accounts for the irregularities of coarse aggregate shapes. By conceptualizing concrete as a solid–liquid biphasic system of mortar and coarse aggregate, we simulate the flow dynamics of concrete within the conveying pipeline and subsequently evaluate pipeline wear and dynamic responses. This research not only introduces a fresh perspective from a methodological standpoint but also underscores the academic significance of systematically studying wear and its influencing factors. Through this integrated approach, scholars can delve deeper into the wear mechanisms during the concrete pumping process, especially under intricate flow conditions, thereby offering insights and guidance to optimize concrete pumping efficiency in practical engineering scenarios.

## Numerical model theory

### Overview of CFD fluid phase

The process of fluid flow and heat transfer follows the laws of mass conservation, momentum conservation and energy conservation, and their mathematical models are continuity equation, Navier–Stokes equation and energy conservation equation, respectively^21^. They are as follows:

1 $$\begin{array}{c}\frac{\partial {\alpha }_{f}{\rho }_{f}}{\partial t}+\nabla \cdot \left({\alpha }_{f}{\rho }_{f}{U}_{f}\right)=0,\end{array}$$

2 $$\begin{array}{c}\frac{\partial }{\partial t}\left({\alpha }_{f}{\rho }_{f}{U}_{f}\right)+\nabla \cdot \left({\alpha }_{f}{\rho }_{f}{U}_{f}{U}_{f}\right)= \,-\nabla {p}_{f}+\nabla \cdot {\alpha }_{f}\tau +{\alpha }_{f}{\rho }_{f}g+{F}_{pf},\end{array}$$

3 $$\begin{array}{c}\frac{\partial }{\partial t}\left({\alpha }_{f}{\rho }_{f}T\right)+\nabla \cdot \left({\alpha }_{f}{\rho }_{f}{U}_{f}T\right)=\nabla \cdot \left(\frac{{k}_{T}}{{c}_{P}}\nabla T\right)+{s}_{T},\end{array}$$

where *α*_*f*_, *ρ*_*f*_, U_*f,*_ and *p*_*f*_ are the volume fraction, density, velocity vector, and pressure component of the fluid, respectively. τ is the viscous stress tensor; *g* is the local gravity acceleration, taking the number of 9.81 m/s^2^; *F*_*pf*_ is the force exerted by all the rock slag on the fluid; *T* is the temperature, *k*_*T*_, and *c*_*P*_ are the heat transfer coefficient and specific heat capacity of the fluid; and *s*_*T*_ is the viscous consumption phase.

In nature, fluids can be divided into Newtonian fluids and non-Newtonian fluids. Water and air are the most common Newtonian fluids. Fluids such as propolis, asphalt, and concrete are non-Newtonian fluids, which often show different rheological properties from Newtonian fluids, and their rheological equations are also different. The commonly used rheological models of non-Newtonian fluids include the H–B model, power-law model, and Bingham model, and Bingham rheological model is adopted in this Paper^22^. The Bingham model can be considered a simplified representation of fluid characteristics to some extent. While there are more precise models available, such as the H–B model, the Bingham fluid model is deemed appropriate when considering computational efficiency and the practicality of the model. The rheological equation is as follows:

4 $$ \begin{array}{*{20}c} {\tau = \tau_{0} + \mu \times \dot{\gamma }\tau \ge \tau_{0} ,} \\ \end{array} $$

5 $$ \begin{array}{*{20}c} {\dot{\gamma } = 0\tau < \tau_{0} .} \\ \end{array} $$

Among them, the yield strength is 270 Pa and the plastic viscosity $\mu$ is 10 Pa·s^20^.

Because of the existence of large irregular stones and high flow rate, the flow of concrete belongs to typical turbulence. The two-equation k–ε turbulence model proposed by Launder and Spalding is recommended to simulate turbulence^23^. The calculation formula is as follows:

6 $$\begin{array}{l}\left\{\begin{array}{l}\frac{\partial \left(\rho \kappa \right)}{\partial t}+\frac{\partial \left(\rho \kappa {u}_{i}\right)}{\partial {x}_{i}}=\frac{\partial }{\partial {x}_{j}}\left[\left(\mu +\frac{{\mu }_{t}}{{\sigma }_{\kappa }}\right)\frac{\partial \kappa }{\partial {x}_{j}}\right]+{G}_{\kappa }-\rho \varepsilon \\ \frac{\partial \left(\rho \varepsilon \right)}{\partial t}+\frac{\partial \left(\rho \kappa {u}_{i}\right)}{\partial {x}_{i}}=\frac{\partial }{\partial {x}_{j}}\left[\left(\mu +\frac{{\mu }_{t}}{{\sigma }_{\varepsilon }}\right)\frac{\partial \varepsilon }{\partial {x}_{j}}\right]+\frac{{C}_{1\varepsilon }\varepsilon }{\kappa }{G}_{\kappa }-{C}_{2\varepsilon }\\ {\mu }_{t}=\rho {C}_{u}\frac{{\kappa }^{2}}{\varepsilon }\end{array}\right.\rho \frac{{\varepsilon }^{2}}{\kappa },\end{array}$$

where *κ* is turbulent energy, *ε* is turbulent dissipation rate, *µ*_t_ is turbulent viscosity, Gκ is turbulence kinetic energy caused by average velocity gradient, and *C*_1*ε*_, *C*_2*ε*_, *C*_*u*_, *σ*_*κ*_, and *σ*_*ε*_ are empirical constants.

### Overview of DEM particle phase

In the process of pipeline transportation, rock slag is affected by other rock slag, mortar and pipe wall, which can be described by Newton’s second law of motion and the discrete element method originally proposed by Cundall and Strack^24^. They are listed as follows:

7 $$\begin{array}{c}{m}_{i}\frac{d{U}_{p,i}}{dt}={m}_{i}g+\sum_{j=1}^{n} \left({F}_{c,ij}+{F}_{d,ij}\right)+\left({F}_{c,iw}+{F}_{d,iw}\right)+{F}_{f,i},\end{array}$$

8 $$\begin{array}{c}{I}_{i}\frac{d{\omega }_{i}}{dt}=\sum_{j=1}^{n}{T}_{ij}+{T}_{iw},\end{array}$$

where *m*_*i*_, *U*_*p,I*_,* I*_*i*_, and *ω*_*i*_ are the mass, velocity, inertial motion and angular velocity of rock slag *i*, respectively. *F*_*c,ij*_, *F*_*d,ij*_, and *T*_*ij*_ are the contact force, viscous damping force and torque between the slag *i* and other slag respectively. *F*_*c,iw*_, *F*_*d,iw*_, and *T*_*iw*_ are the contact force, viscous damping force and torque between the rock slag *i* and the inner wall of the slurry discharge pipe respectively. ***F***_*f,i*_ is the force of fluid phase acting on rock slag* i.*

**Figure 1:**
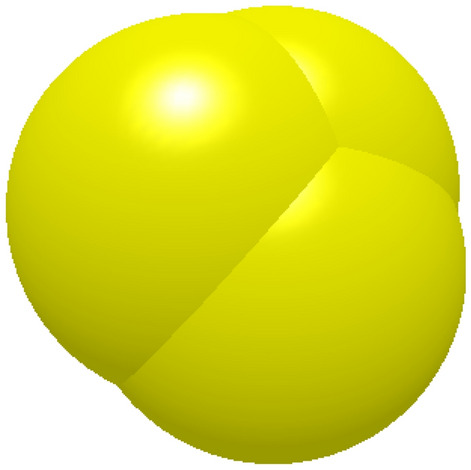
Particle model.

Concrete is composed of coarse aggregate, mortar, admixture and other components, which are different from traditional discrete-phase particles, and its components are more complex. Therefore, when simplifying concrete, it can be roughly divided into coarse aggregate and mortar. Coarse aggregate mainly contains stones with different particle sizes, and mortar mainly contains water, fine aggregate and other materials. In EDEM software, coarse aggregate can be characterized by special-shaped particles, which is more in line with the actual working conditions, as shown in Fig. 1.

Its particle size is expressed by the formula as follows:

9 $$ \begin{array}{*{20}c} {D_{V} = 6V_{S} /\pi^{1/3} .} \\ \end{array} $$

Wherein, ${D}_{V}$ is the equal volume particle size; ${V}_{S}$ is the true volume of the particle.

According to Jiang et al.^20^, three foundation contact Parameters of fresh concrete obtained by simulation are shown in Table 1 below:

**Table 1:** Basic contact parameter values.

Interaction	Static friction coefficient	Coefficient of kinetic friction	Collision recovery coefficient
Particles and particles	0.35	0.015	0.8
Particles and pipes	0.45	0.015	0.8

Common contact models are JKR model, Bond model and Hertz no slip model. In this Paper, Hertz no slip contact model is selected because of the coupling method of CFD-DEM.

In order to explore the wear of conveying pipe, this Paper adopts the built-in wear model of EDEM software, that is, Archard-wear model. The idea of this model comes from the fact that the amount of material removed from the surface is proportional to the friction work done by particles moving on the surface, and its wear equation is as follows^25^:

10 $$\begin{array}{c}Q=\frac{K}{H}{F}_{n}{d}_{t},\end{array}$$

where Q represents the volume of material, ${F}_{n}$ is the normal force, and K is the dimensionless constant, which is determined by the influencing factors of material wear. For different wear problems, it needs to be determined by wear experiments, ${d}_{t}$ is the tangential distance and H is the hardness.

### Interaction between particles and fluid

The interaction force between coarse aggregate and mortar is mainly composed of buoyancy *F*_*B*_, drag *F*_*D*_ and pressure gradient *F*_*P*_, and the formula for calculating the equal force of coarse aggregate is as follows:

11 $$\begin{array}{c}{F}_{B}=-\frac{{\rho }_{f}}{{\rho }_{p}}g,\end{array}$$

12 $$\begin{array}{c}{F}_{D}=\frac{18{\mu }_{e}}{{\rho }_{p}{d}_{p}^{2}}\frac{{C}_{D}R{e}_{p}}{24}\left({U}_{f}-{U}_{p}\right),\end{array}$$

13 $$\begin{array}{c}{F}_{P}=-\frac{1}{{\rho }_{p}}\nabla {p}_{f},\end{array}$$

where *U*_*p*_ is the speed of coarse aggregate, *µ*_*e*_ is the effective viscosity of fluid, *ρ*_*p*_ is the density of coarse aggregate, and *d*_*p*_ is the particle size of coarse aggregate. The Reynolds number *Re*_*p*_ and drag coefficient *C*_*D*_ of coarse aggregate are calculated as follows:

14 $$\begin{array}{c}R{e}_{p}=\frac{\rho {d}_{p}}{\mu }\left|{U}_{f}-{U}_{p}\right|,\end{array}$$

15 $$\begin{array}{c}{C}_{D}={a}_{1}+\frac{{a}_{2}{ }^{2}}{Re}+\frac{{a}_{3}}{Re},\end{array}$$

where *Re* is the Reynolds number of the fluid; *a*_1_, *a*_2_, *a*_3_ are constants, defined by Morsi and Alexander^26^. They are as follows:

16 $$\ a_{1} ,a_{2} ,a_{3}  = \left\{ {\begin{array}{*{20}l}    {0,24,0} \hfill &  {0<Re<0.1}\\ {3.690,22,0.0903} \hfill & {0.1 < \text{Re}  < 1} \hfill  \\    {1.222,29.1667, - 3.8889} \hfill & {1 < \text{Re}  < 10} \hfill  \\    {0.6167,46.50, - 116.67} \hfill & {10 < \text{Re}  < 100} \hfill  \\    {0.3644,98.33, - 2778} \hfill & {100 < \text{Re}  < 1000} \hfill  \\    {0.357,148.62, - 47500} \hfill & {1000 < \text{Re}  < 5000} \hfill  \\    {0.46, - 490.546,578700} \hfill & {5000 < \text{Re}  < 10000} \hfill  \\    {0.5191, - 1662.5,5416700} \hfill & {\text{Re}  \ge 10000} \hfill  \\   \end{array} } \right.. $$

### Coupling model boundary conditions

**Figure 2:**
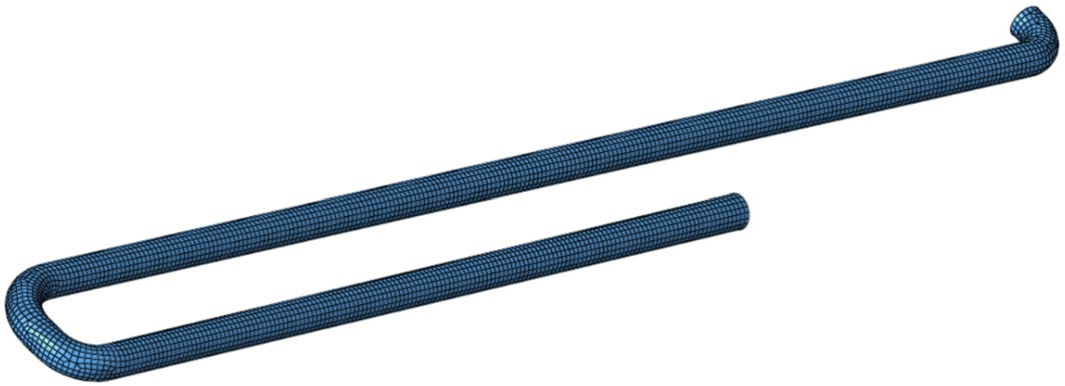
Mesh model of conveying pipe.

In this Paper, Hypermesh software is used to mesh the geometric model of transportation pipeline, and the global mesh size is set to 20 mm^27^, the number of divided grids is 8641, as shown in Fig. 2 below:

In fluent software, according to the actual pumping condition of concrete, the fluid density is 2100 kg/m^3^, the inlet condition is set to 3 m/s, the turbulence is set to intensity and Hydraulic Diameter, the turbulence intensity is 5, and the hydraulic diameter is set to 0.133 m. The outlet condition is set as pressure outlet, and the pressure value is 0, that is, it is connected with atmospheric pressure, and the turbulence setting is the same as the inlet condition. The wall attribute is set to non-slip wall to simplify the calculation. Transient calculation is adopted in the computing environment. Considering the gravity acceleration in the y axis direction, the value is 9.81 m/s^2^. We select standard k-epsilon model.

For EDEM software, the shear modulus of particles is 1e+08 Pa, the density is 2600 kg/m^3^, and Poisson’s ratio is 0.25. The Young’s modulus of the pipeline is 2.1e+11 Pa, the density is 7850 kg/m^3^, and Poisson’s ratio is 0.3. A circular particle factory is set at the inlet of the conveying pipeline, with the particle generation rate of 16.2547 kg/s, the initial particle velocity of 3 m/s and the particle diameter of 5–10 mm.

The setting of time step in CFD-DEM coupling calculation has an important influence on the accuracy of calculation results. The time step of fluid is generally 10–100 times that of solid^28^. In this Paper, the fluent time step is set to 1 $\times {e}^{-4}$ s, and the EDEM time step is set to 1 $\times {e}^{-6}$ s.

## Analysis of coupling calculation results

### Pipeline wear analysis

**Figure 3:**
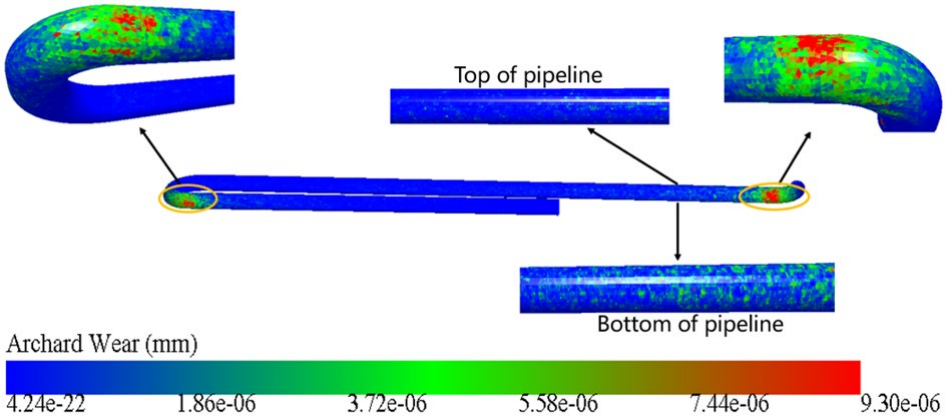
Main wear positions of conveying pipe.

**Figure 4:**
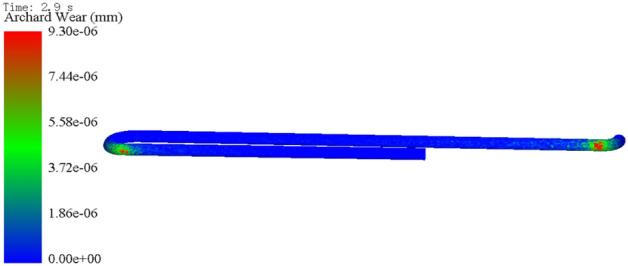
Nephogram of pipeline wear at 2.9 s.

Figure 3 shows the main wear position of the concrete pipeline. It is obvious from the figure that the wear of the conveying pipe mainly occurs at the elbow, especially at the outer side of the elbow. From the gravity direction, the wear position is mainly concentrated at the bottom, and the wear at the top is relatively less serious. The reason for this result is that the mortar carries the solid particles along the streamline. Due to the inertia of the particles, the movement direction of the solid particles at the elbow has changed greatly, while the movement direction of the solid particles at the horizontal straight pipe has not changed, so the solid particles collide with the outside of the elbow earlier, and the movement direction of the particle’s points to the outside of the elbow and backs to the inside of the elbow. This leads to a steady stream of solid particles hitting the outside of the elbow, which makes the wear on the outside of the elbow more serious than that on the inside. In the process of pumping, solid particles also tend to move and deposit to the bottom of the conveying pipe due to gravity, resulting in greater wear at the bottom of the conveying pipe than at the top. Figure 4 shows the wear nephogram of the conveying pipe when pumping for 2.9 s. The maximum wear in the nephogram is 9.3e−6 mm. Because the wall thickness of the conveying pipe is 7 mm, it can be roughly calculated that the service life of the conveying pipe is 606 h. In reality, the service life of concrete pump truck conveying pipes is approximately 600–700 h, with an average of 650 h. Compared to this, the error in our results is a mere 6.77%, further attesting to the accuracy of our simulation approach.

**Figure 5:**
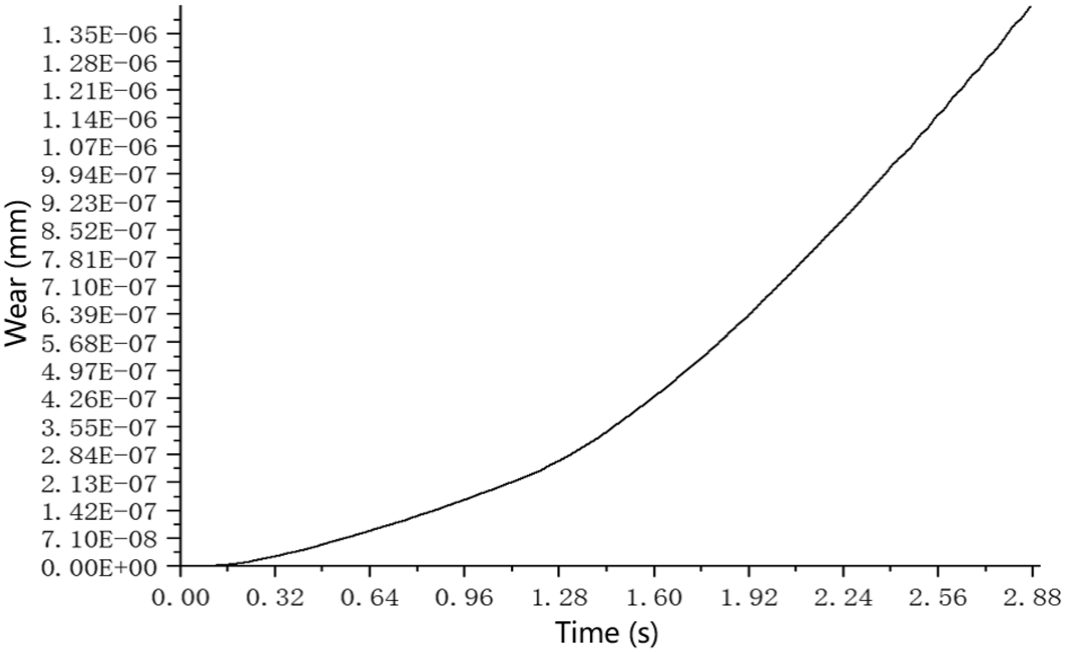
Graph of average wear amount with time.

Figure 5 is a graph showing the change of the average wear amount of the conveying pipe with time. It can be seen from the graph that the wear amount of the conveying pipe increases in the form of a quadratic function with the increase of time. This is attributed to the particles within the concrete mixture undergoing both impact and frictional interactions with the pipeline walls during conveyance, with both mechanisms contributing to wear. The magnitude of the impact force from the particles is determined by their velocity, mass, and angle of incidence against the pipeline wall. On the other hand, the frictional force is a function of the contact area between the particles and the wall, coupled with the coefficient of friction. As the wear on the pipeline surface intensifies, it becomes increasingly abrasive, elevating the frictional interactions between the particles and the wall. This, in turn, accelerates the wear process. However, as time progresses, the amplifying effect of particle impact starts to wane, leading to a deceleration in the wear rate, culminating in a quadratic growth Pattern. In addition, the wear amount hardly increases at 0–0–0.16 s. From 0.16 to 1.28 s, the wear amount of the conveying pipe begins to increase slowly with the increase of time, and after 1.28 s, the wear amount increases sharply compared with before. From the trend of the curve, it can be predicted that if the concrete is pumped continuously, the wear amount will increase according to the wear speed after 1.28 s until the pipeline is worn out.

### Analysis of motion characteristics

**Figure 6:**
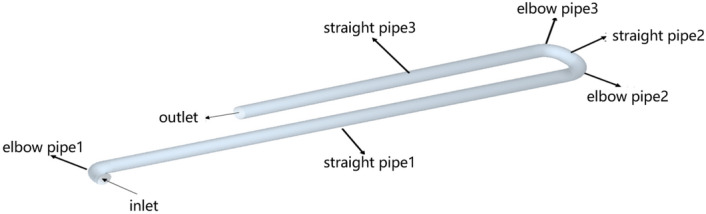
Schematic diagram of each Part of the conveying pipeline.

In order to describe the movement state of concrete particles, all Parts of the conveying pipeline are named in this Paper, as shown in Fig. 6 below.

**Figure 7:**
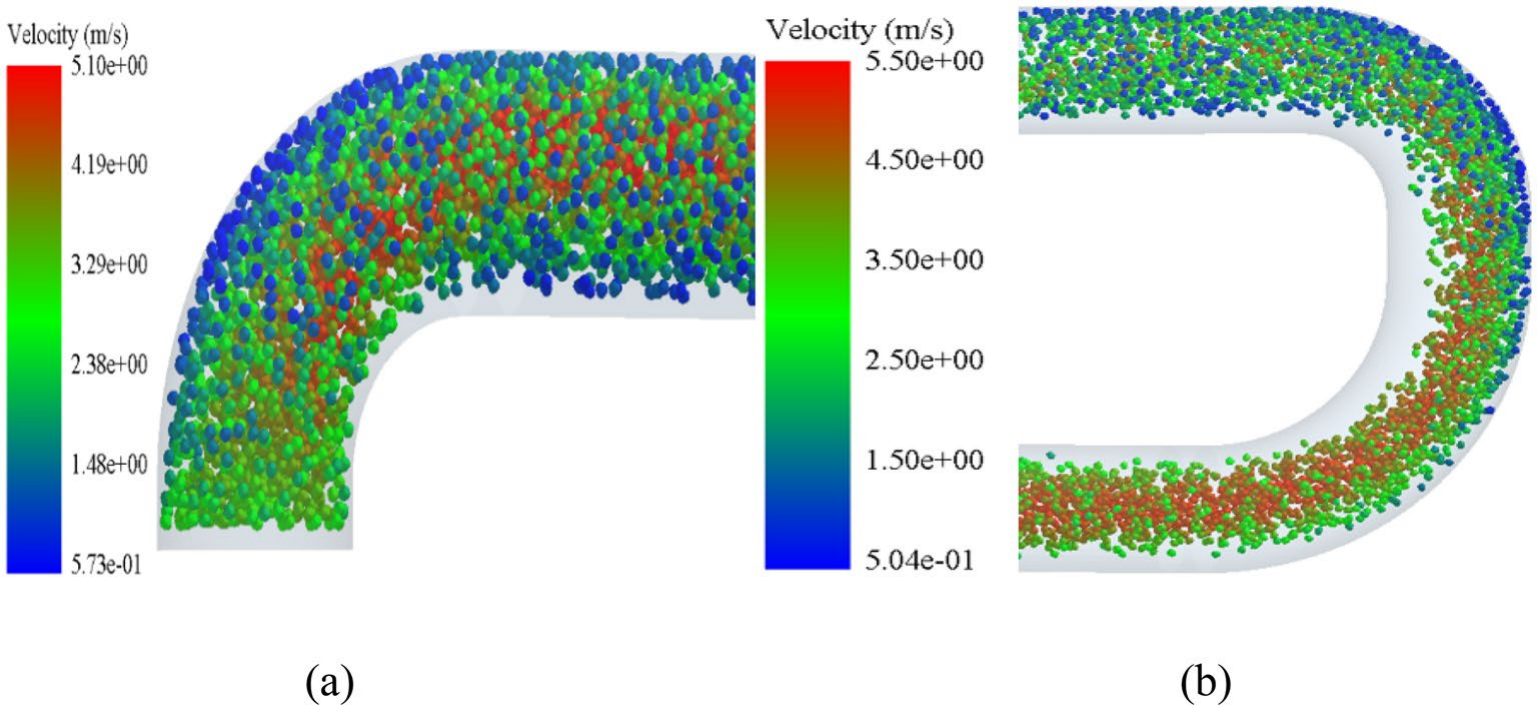
Nephogram of particle velocity at various parts of the conveying pipe: (**a**) Elbow 1 from above, and (**b**) Elbow 2–Elbow 3 from above.

**Figure 8:**
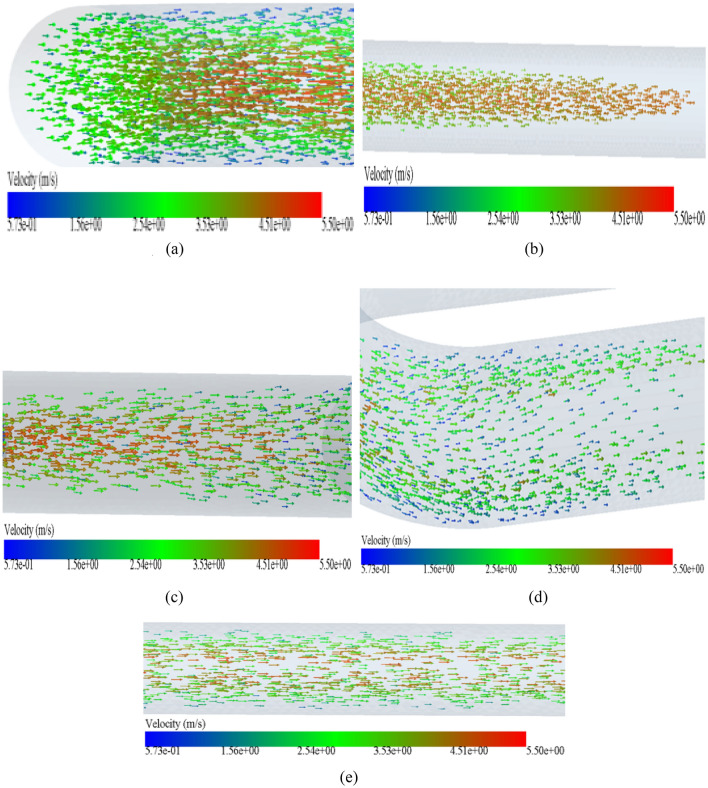
The particle velocity vector diagram of each part of the conveying pipe: (**a**) the elbow 1 in the front view, (**b**) the straight pipe 1 in the front view, (**c**) the straight pipe 2 in the front view, (**d**) the elbow 3 in the side view, and (**e**) the straight pipe 3 in front view.

Figure 7a is a nephogram of the particle velocity when overlooking the elbow 1. It can be seen from Fig. 7a that when concrete particles are pumped from the inlet of the conveying pipe, the particle velocity in the central Part of the pipe increases slightly comPared with the initial one when passing through the elbow pipe 1. It shows a trend that the particle velocity in the central Part is the largest, and the closer it is to the pipe wall, the lower it is, because the particles in the central area are not rubbed and collided by the pipe wall. For the inner wall of elbow pipe 1, when particles enter straight pipe 1 from elbow pipe 1, the velocity of particles near the inner wall of the pipe decreases. Figure 8a is a particle vector diagram of elbow 1. From Fig. 8a, it can be seen that the particles in the central Part of elbow 1 move in the axial direction of the pipe, but the particles near the pipe wall move in a somewhat irregular direction, which is due to the interaction between particles near the pipe wall and the pipe, resulting in the trend of jumping up and down. Figure 8b is a particle vector diagram when the straight pipe 1 is viewed from the front. When the particles enter the straight pipe 1 from the bent pipe 1, it can be seen from Fig. 8b that the particle moving in the front is the fastest, and the moving direction of the particles in the straight pipe 1 is more regular, because the moving direction of the particles in the horizontal straight pipe is consistent with the extending direction of the pipe, and there is no big impact collision with the pipe. Figure 7b is a nephogram of particle velocity when looking down at elbow pipe 2-elbow pipe 3. When particles move from elbow pipe 2 to elbow pipe 3, it can be seen from Fig. 7b that the overall velocity of particles is greatly reduced. Moreover, due to the high velocity of the pipeline in a stable state, there is a transition phenomenon when particles Pass through a 90-degree elbow pipe, resulting in almost no particles near the inner wall of the pipeline, and particles all move along the outer side wall surface of the pipeline, which makes the outer side wall surface of the elbow wear more than others. Figure 8c is a particle vector diagram when the straight pipe 2 is viewed directly. When the particles Pass through the straight pipe 2, it can be clearly seen from Fig. 8c that the motion of the particles is no longer consistent along the axial direction of the pipe, but begins to spread up and down to the pipe wall, jumping and rolling around. Figure 8d is a particle vector diagram when elbow pipe 3 is viewed from the side. When the particles enter the elbow pipe 3, it can be seen from Fig. 8d that the particle distribution is no longer aggregated in the middle, but decreased at the upper and lower sides, but the number of particles in the central area is reduced, and the particles near the pipe walls at the upper and lower sides are aggregated. Figure 8e is a particle vector diagram when straight pipe 3 is viewed from the front. After the particles move to the straight pipe 3, it can be seen from Fig. 8e that the motion of the particles returns to the same motion state as that of the horizontal straight pipe 1.

**Figure 9:**
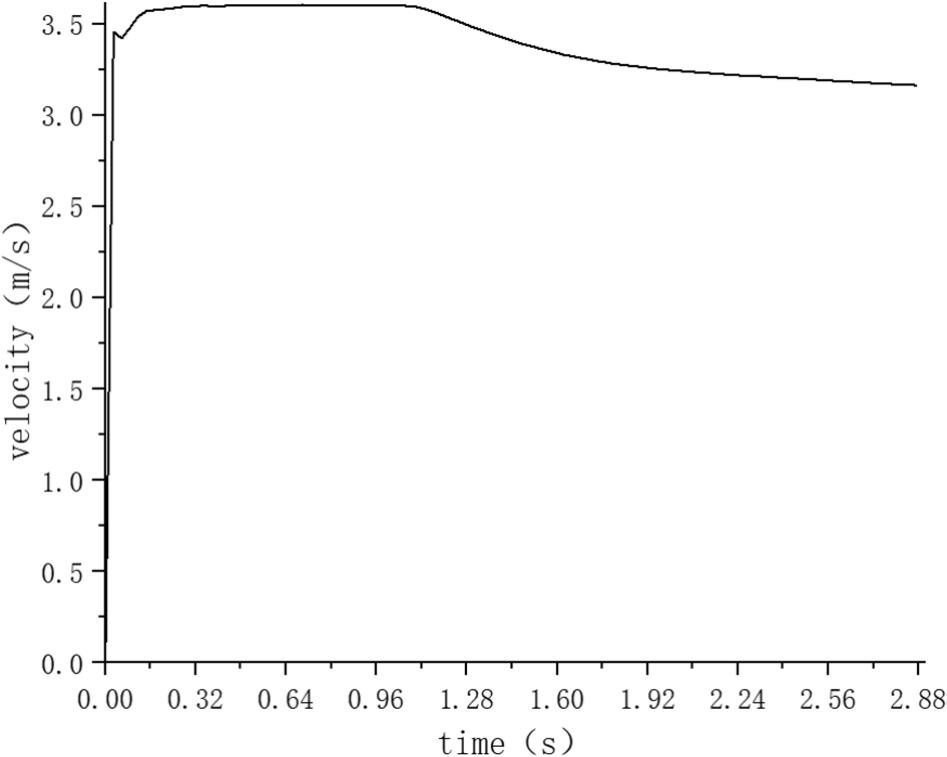
Variation of mean particle velocity with time.

Figure 9 is a curve chart showing the change of average particle velocity with time. It can be seen from Fig. 9 that the particle velocity soared rapidly in a very short time, and exceeded the initial velocity at the entrance. This is because the unique geometry of elbow pipe 1 induces the formation of vortices and turbulence as the fluid traverses. This fluid dynamic phenomenon intensifies the interplay between particles and the fluid, culminating in an accelerated particle velocity. Additionally, the fluid’s velocity within the bend might momentarily surpass the initial speed at the entrance, further amplifying the particle acceleration. Interactions amongst particles, such as collisions and friction within the elbow, also contribute to the velocity surge, and then the average particle velocity remained stable at 3.61 m/s after reaching the horizontal straight pipe 1. When it reached 1.16 s, the particle velocity decreased greatly, because the particles began to enter the elbow pipe 2 and the elbow pipe 3, and finally the average particle velocity gradually stabilized at 3.15 m/s. Compared with the initial pumping speed, the particle velocity after pumping stability increased by 5%. The increment in velocity suggests that concrete can be delivered to its intended location in a reduced timeframe, enhancing operational productivity. However, looking at wear Patterns, the escalated particle velocity might result in accelerated wear on the pipeline walls, particularly in regions susceptible to recurrent particle–wall collisions. Thus, in practical concrete pumping scenarios, there may be a necessity to refine pumping strategies and to conduct more frequent pipeline maintenance and replacements, ensuring consistent operational efficiency while extending the pipeline’s longevity.

**Figure 10:**
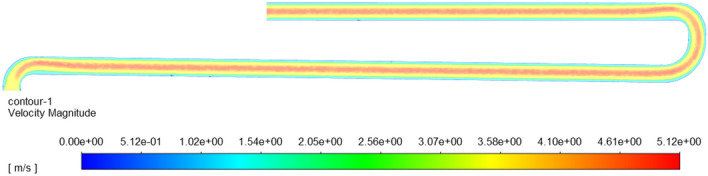
Nephogram of axial fluid velocity in pipeline.

**Figure 11:**
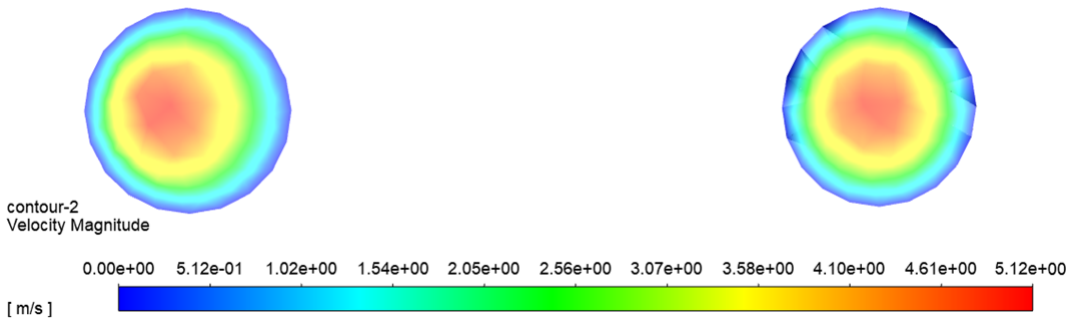
Nephogram of fluid velocity in radial section of pipeline.

Figure 10 is a nephogram of fluid velocity in the axial central section of the conveying pipe, and Fig. 11 is a diagram of fluid velocity in the radial section between the straight pipe 1 and the elbow pipe 2 and between the elbow pipe 3 and the straight pipe 3. From Figs. 10 and 11, it can be seen that the velocity of fluid is the largest in the central area of the pipeline, and it gradually decreases from the middle to the pipe wall, and the velocity of fluid close to the pipe wall is almost zero, because the closer it is to the pipe wall, the greater the friction the fluid receives, and the fluid itself is viscous, so the velocity of fluid close to the wall is zero. It can also be seen from Fig. 11 that the fluid in the left radial cross section is generally biased to the left, while the fluid in the right radial cross section is more evenly distributed. This is because the fluid in the left radial cross section is behind the elbow pipe 3 and is subjected to inertia when Passing through the elbow pipe 3, and the fluid is generally biased to the left outer wall, while the fluid in the right radial cross section is in the middle of the horizontal straight pipe 1, so the fluid movement is relatively stable. Compared with the movement speed of particles, the fluid velocity is larger, which accelerates the movement of particles.

### Analysis of influencing factors

**Figure 12:**
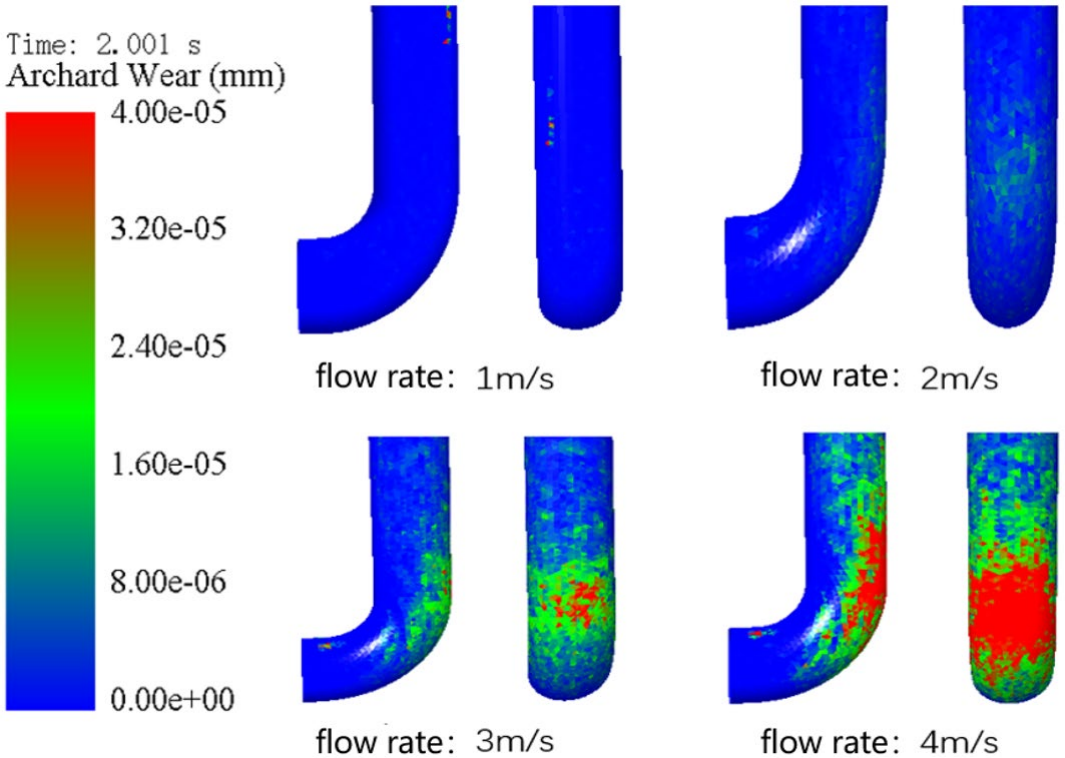
Wear nephogram of the outer side of elbow at different flow rates.

**Figure 13:**
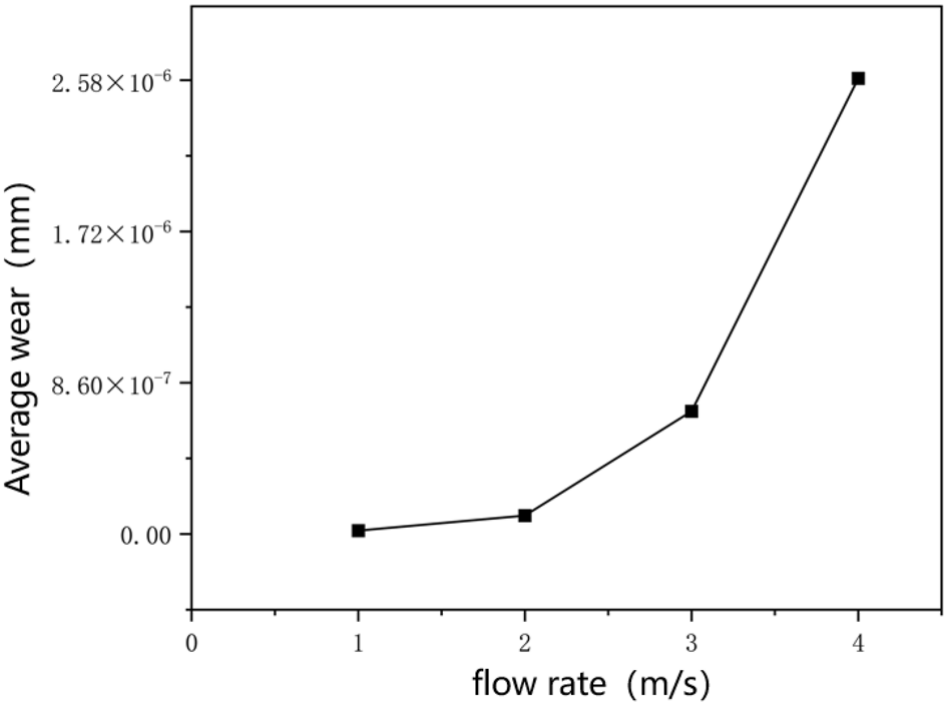
Relationship between velocity and average wear of pipeline.

Firstly, this study investigates the influence of varying pumping speeds on pipeline wear. When the particle volume fraction is 15%, the solid particle size is 8 mm, and the time is 2 s, the pipeline wear is simulated and analyzed at different initial pumping speeds, in which the initial flow rates are set to 1 m/s, 2 m/s, 3 m/s and 4 m/s, respectively. Figure 12 shows the wear nephogram of elbow with different initial pumping speeds when the maximum wear amount is 4e−5 mm. It can be seen that the greater the flow rate, the larger the area above the maximum wear amount. When the flow rate is lower than 3 m/s, the change of the wear area is not obvious, while when the flow rate is higher than 3 m/s, the wear area is obviously enlarged. Therefore, it can be seen that the optimal pumping speed should be between and 3 m/s. When the concrete is pumped for 2 s, the influence of different pumping speeds on the average wear of the conveying pipeline is shown in Fig. 13. It can be seen that the relationship between the flow speed and the average wear is similar to exponential or quadratic, but nonlinear. The greater the flow speed, the more intense the average wear will increase. After calculation, when the pumping speed is 2 m/s, the wear increment is 5 times that of 1 m/s. When the pumping speed is 3 m/s, it becomes 35 times. When the pumping speed reaches 4 m/s, it quickly becomes 135 times, which is roughly the same as the intuitive conclusion from the wear cloud map.

**Figure 14:**
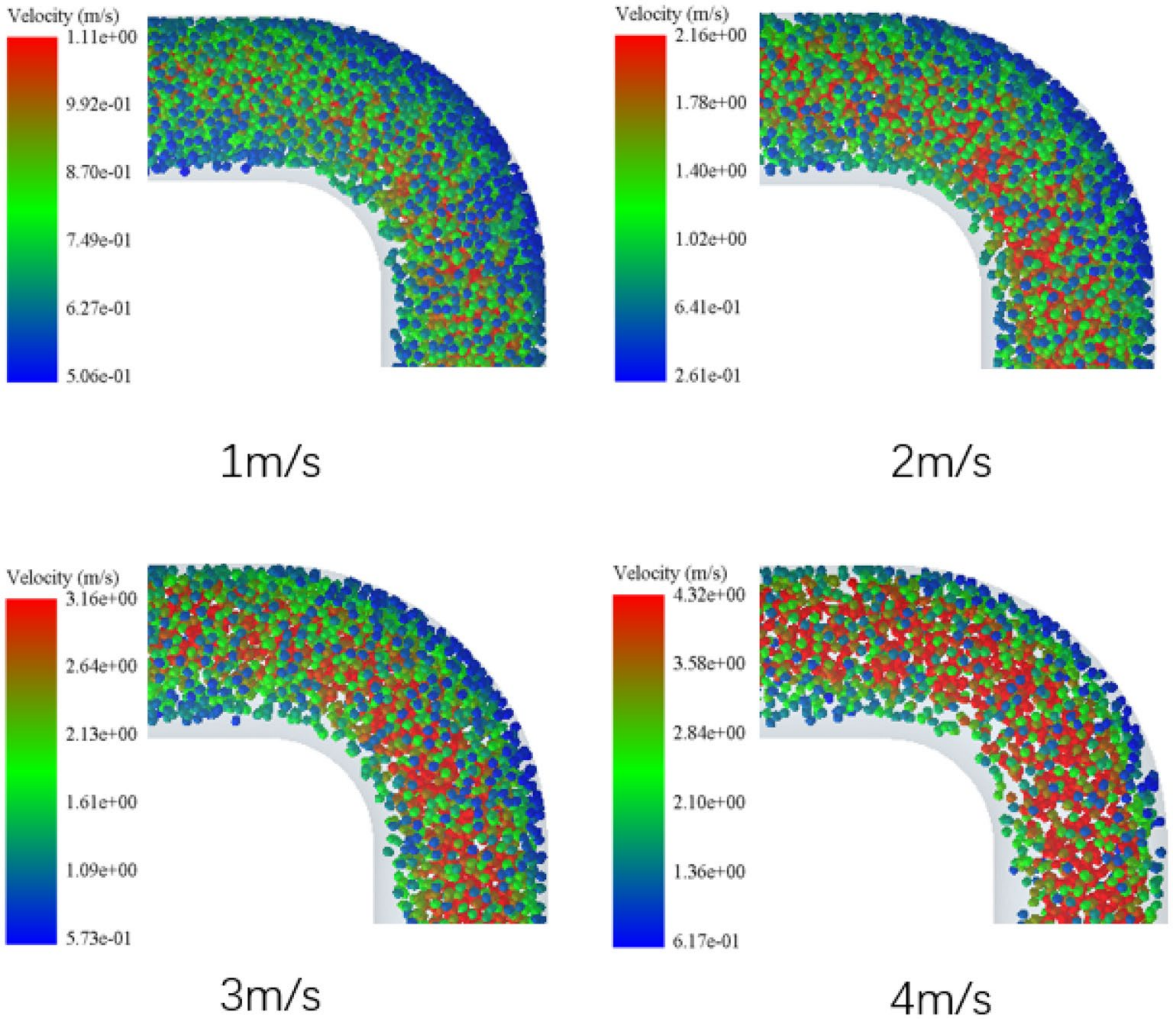
Particle velocity nephogram of elbow 3 at different flow rates.

From the above analysis of motion characteristics, it is concluded that the motion state of solid particles will change dramatically when they Pass through the elbow 3, which is the most obvious change compared with other positions of the conveying pipe. Figure 14 shows the nephogram of particle velocity of elbow pipe 3 under the conditions of flow velocity of 1 m/s, 2 m/s, 3 m/s and 4 m/s respectively. It can be clearly seen from the figure that the lower the flow velocity, the more serious the elbow blockage is. When the flow velocity is 4 m/s, the distribution of particles in elbow 3 is sparse and the number is relatively small. When the flow velocity is 3 m/s, the number of particles in elbow pipe 3 has obviously increased, and the particle accumulation phenomenon has begun to appear on the outer wall of elbow, and the density is higher than before. When the flow velocity is 1 m/s, the particles are densely accumulated on the outer wall of elbow pipe, and the elbow blockage is very serious. Particle accumulation can lead to increased pumping pressures, thereby impacting the efficiency of the pumping process. Concurrently, the accumulation of particles intensifies wear within the pipeline, especially in regions with significant accumulation, potentially reducing the pipeline's operational lifespan. Furthermore, such accumulation results in the stratification of the concrete, compromising its uniformity and overall quality. Through comprehensive consideration, at pumping speeds exceeding 3 m/s, the pipeline experiences substantial wear. At speeds below 2 m/s, there's a marked decrease in pumping efficiency accompanied by significant particle accumulation. However, within the range of 2 m/s to 3 m/s, pumping efficiency is optimized, wear is minimized, and particle accumulation is less pronounced, so it is most appropriate to set the concrete pumping speed within the range of 2–3 m/s.

**Figure 15:**
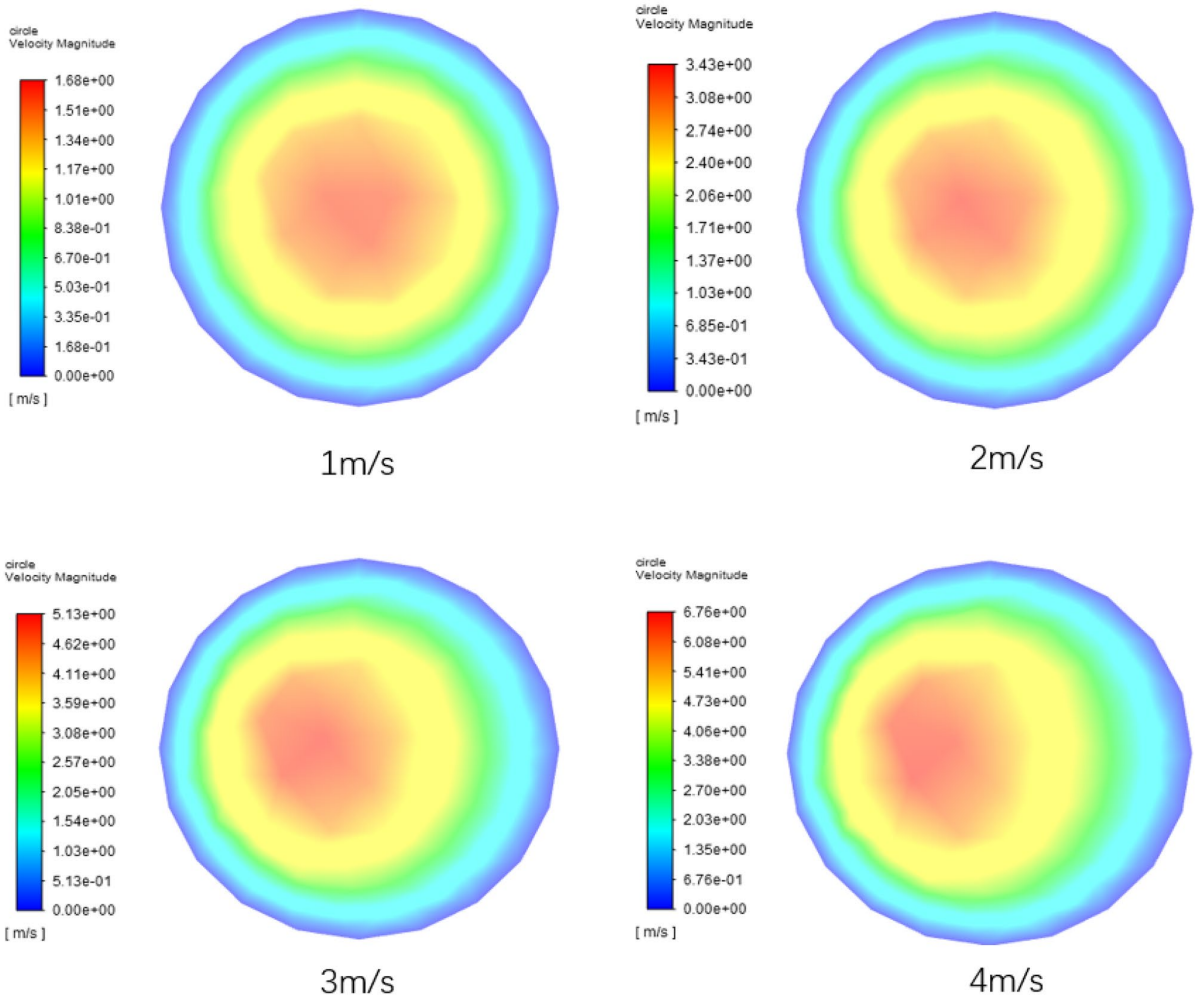
Velocity nephogram of radial section at different initial velocities.

Figure 15 is a nephogram of the fluid velocity between the elbow pipe 3 and the straight pipe 3 (near the elbow pipe 3, with the outer wall on the left side of the view) when the time is 2 s. From this figure, it can be seen that when the initial velocity is 1 m/s, the velocity equivalent area in the nephogram is evenly distributed in a concentric circle shape, and with the continuous increase of the initial velocity, the velocity equivalent area tends to the left side (outside the pipe wall). However, in the direction of gravity, the velocity area is evenly distributed, always in the center position, and the eccentricity phenomenon is not obvious. This is attributed to the fact that Elbow 3 possesses a 90° bend in the direction perpendicular to gravity. This curvature results in the fluid, due to inertia, skewing towards the external side of the bend (visible as the left side in the sectional view). Given that the spatial orientation of Elbow 3 is horizontally aligned, meaning it has a 0° inclination concerning the gravitational axis, the fluid traversing through Elbow 3 is unaffected by gravitational forces. Consequently, there's a uniform distribution of flow velocity in the gravitational direction.

**Figure 16:**
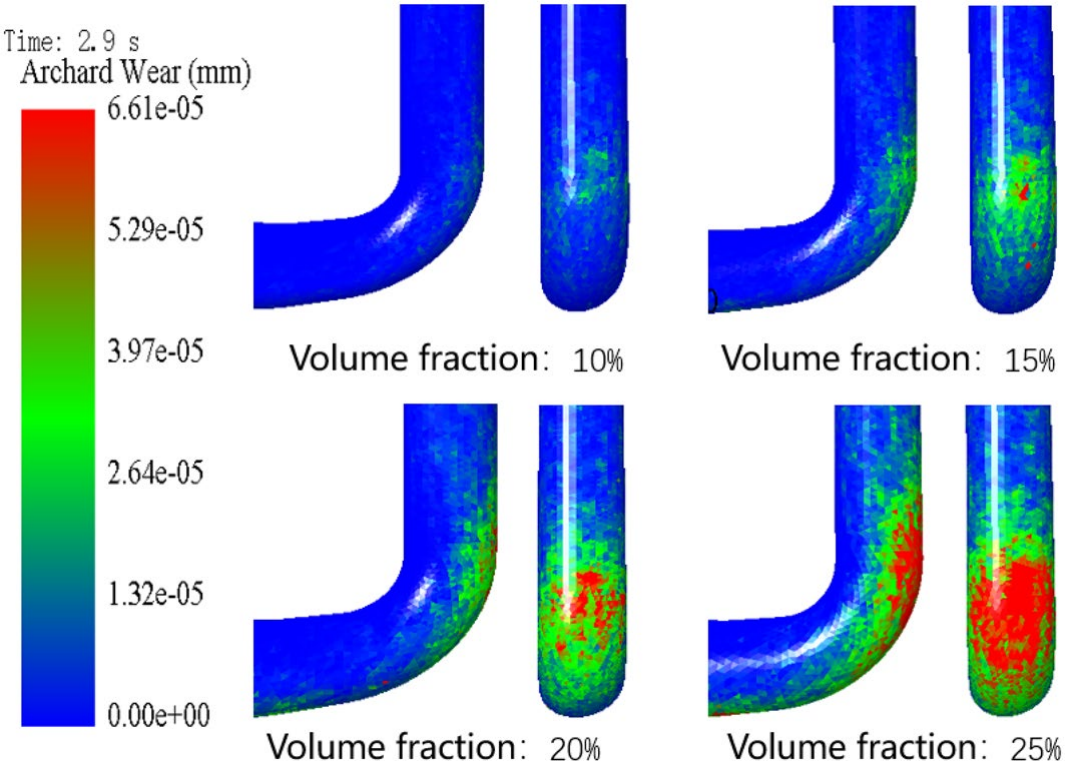
Wear nephogram of the outer side of elbow pipe with different volume fraction of coarse aggregate.

**Figure 17:**
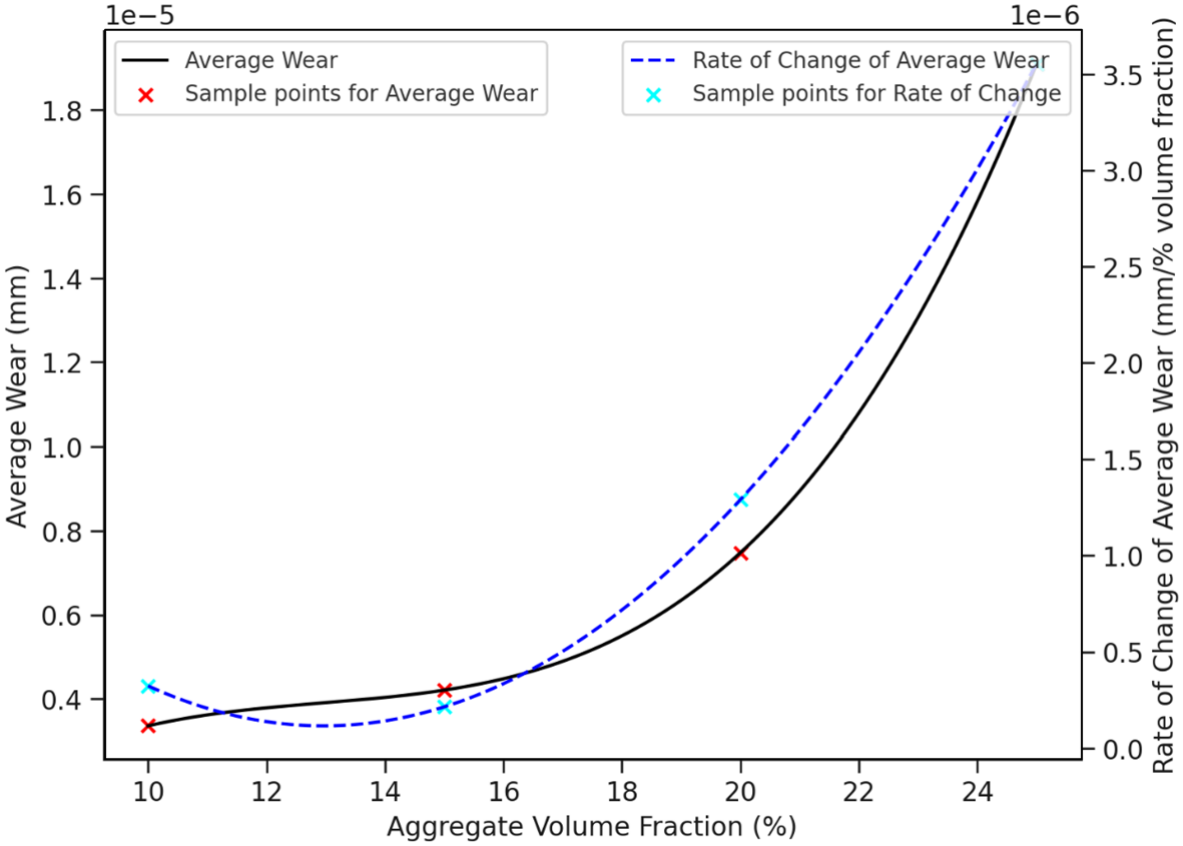
Relationship of coarse aggregate volume fraction to pipe average wear and its rate of change.

Subsequently, the study delves into the impact of different coarse aggregate volume fractions on pipeline wear. When the pumping speed is 3 m/s, the particle size of coarse aggregate is 8 mm and the simulation time is fixed at 2.9 s, the wear of conveying pipes with different volume fractions of coarse aggregate is numerically simulated and analyzed. The generation rate of coarse aggregate is related to the volume fraction and initial speed of coarse aggregate, so the volume fraction of coarse aggregate is expressed by changing the generation rate, and the volume fraction of coarse aggregate is set to be 10%, 15%, 20% and 25%, respectively. Figure 16 shows the wear nephogram of the outer side of the elbow with different volume fractions of coarse aggregate when the same wear amount is achieved and the pumping time is 2.9 s. It can be seen that the wear area of the outer side of the elbow pipe increases with the increase of the volume fraction of coarse aggregate. When the volume fraction increases from 20 to 25%, the wear area of the outer side of the elbow pipe expands more obviously, while when the volume fraction increases from 15 to 20%, the wear area of the outer side of the elbow pipe expands relatively slowly. From the above analysis, it is reasonable to set the volume fraction of coarse aggregate to 15–20%, considering that too little volume fraction of coarse aggregate will affect the strength and cohesion of concrete, and too large volume fraction of coarse aggregate will reduce the pumping efficiency of concrete and even lead to the blockage of conveying pipes. Figure 17 is a graph showing the relationship curve of average wear and wear rate in conveying pipes with different volume fractions of coarse aggregate when the time is 2.9 s. It can be seen that with the increase of the volume fraction of coarse aggregate, the average wear of the pipeline increases at the same time. When the volume fraction of coarse aggregate is between 10 and 20%, the average wear of the pipeline increases slowly, while the wear degree increases sharply between 20 and 25%. This is because the volume fraction of coarse aggregate increases, and the number of collisions between coarse aggregate and the wall of the pipeline increases at the same time, resulting in increased wear of the pipeline. The increase in wear rate exhibits a nonlinear accelerating characteristic. As the volume fraction increases, the growth rate of wear also accelerates. This trend is likely since as the particle volume fraction increases, collisions between particles and between particles and the pipe wall become more frequent, leading to higher friction and impact. Moreover, with the rise in volume fraction, there are more solid particles in the fluid, which may make the flow more challenging and intensify the friction and impact force of the fluid and particles on the pipe.

Due to the material properties of concrete, an excessive volume fraction of aggregates can lead to stratification, while a deficient volume fraction can result in segregation and bleeding. Consequently, most existing studies on the variation range of coarse aggregates are conducted within the 10–25% range. Notably, wear is more pronounced at the bend locations in pipelines. The wear is also influenced by different bending angles of the bend pipe. However, even with varying bending angles, current research indicates that the wear rate increases in a quadratic function with the volume fraction, which is consistent with the findings of this study^27^. Furthermore, an increase in the volume fraction reduces the compressive strength, tensile strength, and flexural strength of the concrete^29^.

**Figure 18:**
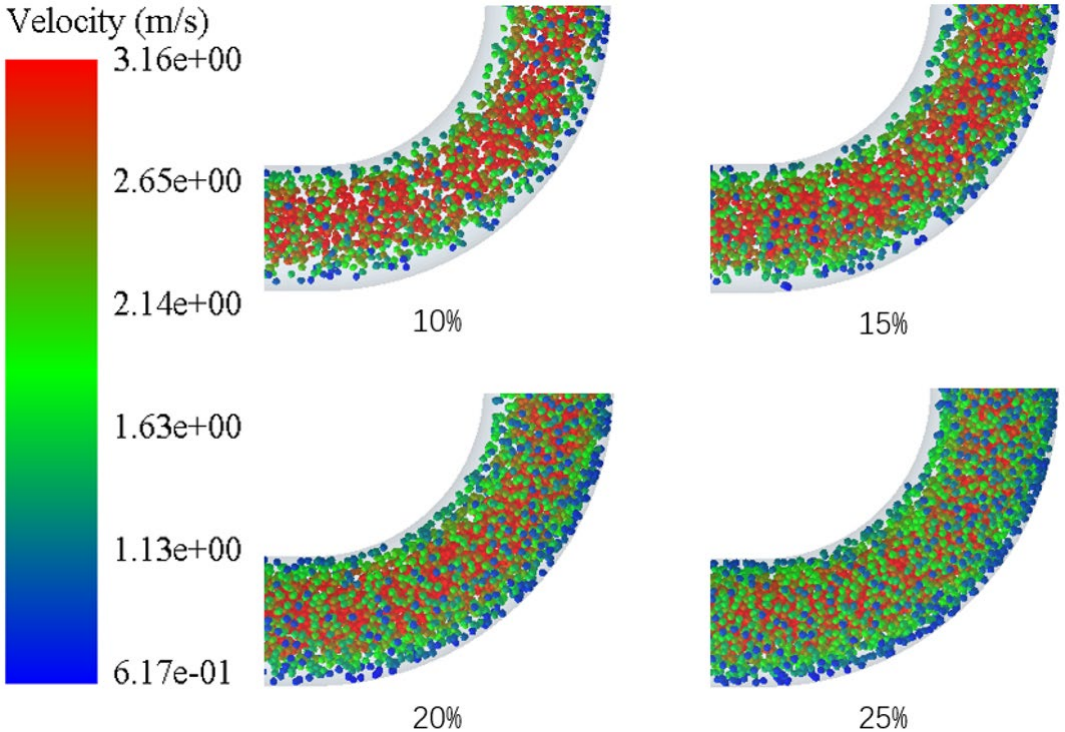
Nephogram of particle velocity of elbow pipe 2 with different volume fraction of coarse aggregate.

Figure 18 is a nephogram of the particle velocity of elbow pipe 2 at 2.9 s when the volume fraction of coarse aggregate is 10%, 15%, 20% and 25%, respectively. It can be seen from the figure that the particle movement is biased to the outside of elbow pipe 2. When the volume fraction of coarse aggregate is 10%, the particle distribution is sparse, but with the increase of volume fraction, the particles gradually become dense. When the volume fraction of aggregate reaches 25%, the whole elbow pipe 2 is almost blocked by aggregate.

**Figure 19:**
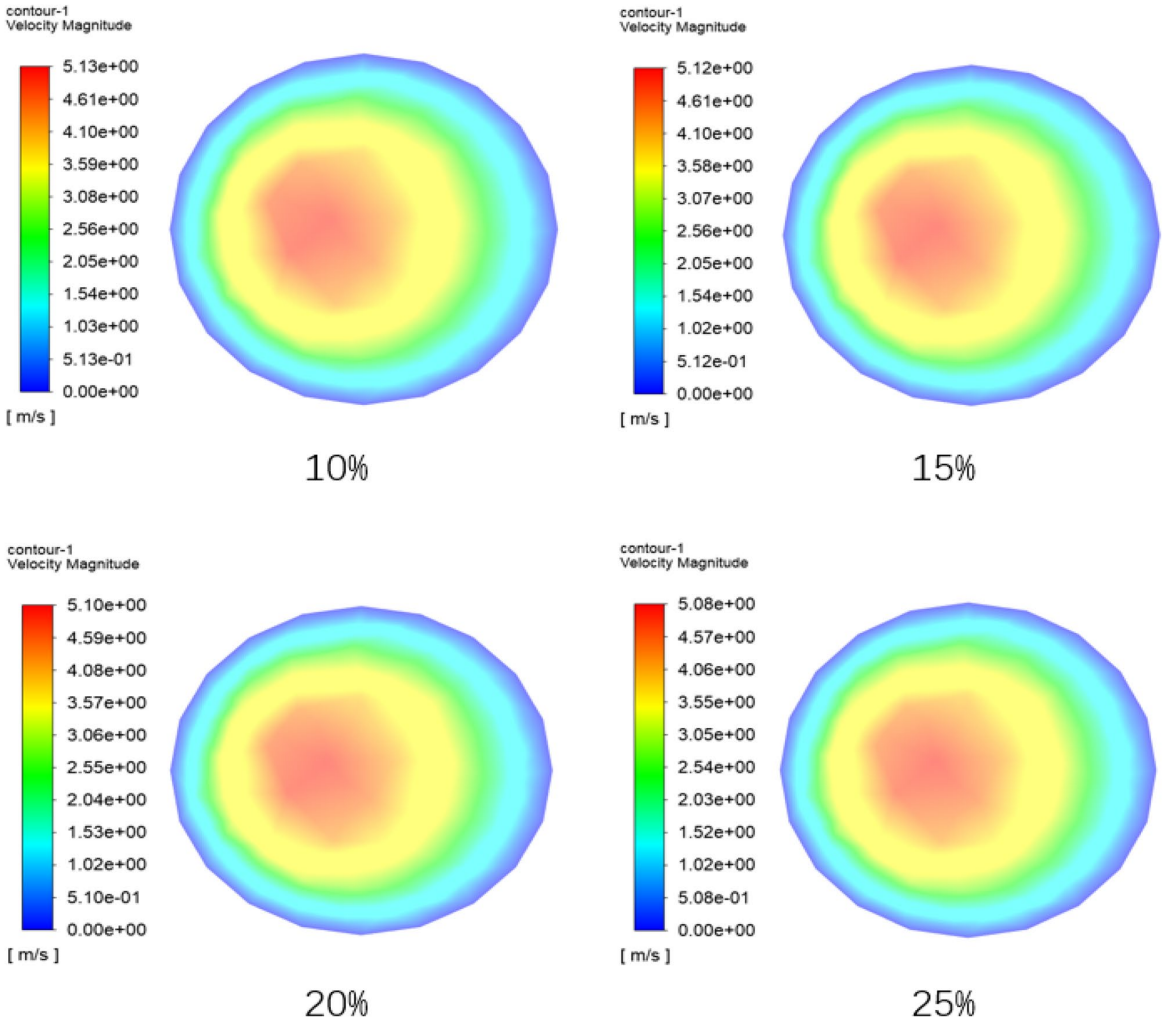
Velocity nephogram of radial section with different coarse aggregate volume fraction.

Figure 19 is a nephogram of the fluid velocity in the radial section between the elbow pipe 3 and the straight pipe 3 (near the elbow pipe 3, the left side of the view is the outer wall) when the time is 2.9 s. It can be seen from the figure that the larger the volume fraction of the coarse aggregate, the smaller the maximum fluid velocity, which is because the coarse aggregate has a hindrance to the fluid flow. The presence of coarse aggregates makes the flow Path of the fluid more intricate. Acting as obstructions within the fluid, coarse aggregates necessitate the fluid to navigate around them, thereby amplifying the complexity of the flow. This not only alters the flow Pattern of the fluid, transitioning from laminar to turbulent, but also heightens the friction between the fluid and the pipeline wall as well as between the aggregates. The more the coarse aggregate content, the greater the hindrance, but the initial pumping speed of the particles is the same under these four working conditions.

## Conclusions

In this study, the CFD-DEM coupling approach was employed, considering the irregularity of coarse aggregate shapes, to simulate the concrete pumping process within conveyance pipes. The specific conclusions are as follows.

1. During the pumping of concrete, the outer side and bottom of the bent pipe exhibit pronounced wear, with the wear amount increasing quadratically over time from 0 to 1.42E−06 mm. Therefore, enhanced inspection of these areas is recommended.
2. In bent pipes, particles predominantly move along the outer wall, whereas their motion in straight pipes is more uniform.
3. The wear amount is correlated with the initial pumping speed and the volume fraction of coarse aggregates. For optimal results, it is recommended that the initial pumping speed be set between 2 and 3 m/s, and the volume fraction of coarse aggregates be maintained at 15–20%.

While this study accounted for the irregularity of coarse aggregates, future research can further investigate the effects of different aggregate materials, shapes, grain sizes, and gradations on pipe wear and pumping efficiency.

## Data Availability

Data will be made available on request. And if you want to get data from this study, please contact corresponding author (Yuankun Liao, 1348638810@qq.com).
